# Dual tension band technique for patellar fractures involving articular surface and inferior pole: a retrospective cohort study and finite element analysis

**DOI:** 10.3389/fbioe.2025.1530745

**Published:** 2025-03-04

**Authors:** Yanchun Gao, Guifa Wu, Hongli Zhu, Kaixin Bian, Di Wu, Haifeng Wei

**Affiliations:** ^1^ Department of Orthopedic Surgery, Shanghai Sixth People’s Hospital, Shanghai Jiao Tong University, Shanghai, China; ^2^ Department of Orthopedic Surgery, Jiuting Hospital, Shanghai, China; ^3^ Department of Technology, Chongqing Optical Machinery Research Institute, Chongqing, China

**Keywords:** patellar fracture, inferior pole of the patella, patella baja, insall-salvati index, finite element analysis

## Abstract

**Background:**

Fractures involving the inferior pole of the patella can lead to postoperative patella baja. For comminuted patellar fractures that affect both the articular surface and the inferior pole, we aim to explore a new fixation method that provides reliable internal stabilization while reducing the incidence of patella baja.

**Methods:**

We conducted a finite element biomechanical study and a retrospective cohort clinical study. The finite element analysis compared the “dual tension band” technique to the traditional single tension band method using 3D models of patellar fractures. The clinical study included 66 patients with patellar fractures involving the articular surface and inferior pole, divided into two groups based on the fixation method. Outcomes were assessed using a range of motion (ROM), Böstman scores, and the Insall-Salvati Index (ISI).

**Results:**

The finite element analysis revealed that the dual tension band technique resulted in lower maximum stress on the patella and lower displacement on the fixation devices compared to the single tension band. Clinically, patients treated with the dual tension band had significantly higher postoperative ISI values (0.93 ± 0.16 vs. 0.85 ± 0.17, p < 0.05), better ROM (123.75 ± 9.58 vs. 117.63 ± 12.28, p < 0.05), and a lower incidence of patella baja (17.86% vs. 34.21%). The Böstman scores showed no significant difference between the groups.

**Conclusion:**

The dual tension band technique provides effective stabilization for patellar fractures involving the articular surface and inferior pole, reducing the incidence of postoperative patella baja and improving functional outcomes.

## 1 Introduction

Patellar fractures are a common injury, accounting for approximately 0.5%–1.5% of all trauma-related fractures (Bostrom, 1972). As an essential component of the knee extension mechanism, the integrity of the patella is crucial for knee function. For many patellar fractures, surgical intervention is often necessary to achieve proper joint surface alignment and fracture healing (Levack et al., 1985; Gao et al., 2023a). Various internal fixation methods have been developed over time, including tension band wiring, patellar plates, patellar claws, non-metallic fixations, and vertical wiring (Kim et al., 2011; Lin et al., 2015; Matejcic et al., 2015; Bonnaig et al., 2015; Malik and Halwai, 2014; Yang and Byun, 2003) However, the increasing demand for functional outcomes has raised the standards for clinical treatment.

Research has shown that postoperative patella baja is associated with knee discomfort and limited function (Paulos et al., 1987; Paulos et al., 1994). These fractures often present challenges due to small fragments, weak cancellous bone, and comminution (Saltzman et al., 1990). For comminuted fractures involving the articular surface and the inferior pole, the choice of fixation method becomes even more critical (Cho et al., 2018; Buschbeck et al., 2022). Effective and reliable internal fixation for comminuted fractures involving the inferior pole of the patella has been pending exploration by surgeons (Zhu et al., 2020; Zhu et al., 2022).

To address this specific type of fracture, we utilized the “dual tension band” fixation technique. Introduced in 2016, this method has been applied to both patellar fractures and revision surgeries following fixation failures (Xue et al., 2016; Zhang et al., 2020). This technique aims to provide effective stabilization while reducing the incidence of patella baja. This paper presents a comparative analysis of this new fixation method against the traditional single tension band technique using finite element models and includes a statistical analysis of retrospective clinical study results to discuss the feasibility and effectiveness of this novel approach.

## 2 Materials and methods

### 2.1 Finite element biomechanical study

A finite element biomechanical study was conducted to evaluate the stability of the “dual tension band” technique. CT data of the patient’s left knee joint in DICOM format were imported into Mimics Research 19.0 medical modeling software. Using commands such as threshold adjustment, region growth, segmentation masks, and mask editing, the patella model was reconstructed and exported as an STL file. This STL file was then imported into Geomagic Wrap 2017 for further refinement, including smoothing, mesh refinement, and high-precision surface construction, and saved in STEP format (Supplemental Materials S1).

The STEP file was imported into NX12.0 for 3D design and modeling. Curves were drawn on the patellar surface and at central locations to simulate intraoperative banding and vertical fixation, with wire diameters set to 1 mm. The patella was then split to simulate a fracture involving the articular surface and inferior pole.

The patellar 3D model was imported into Abaqus 6.14 for finite element analysis. Material parameters, including elastic modulus, Poisson’s ratio, and material density, were defined (Supplemental Materials S2). A dual tension band model was created to treat patellar fractures involving the articular surface and inferior pole. The tension load on the superior patellar pole was gradually increased from 0 to 500 N within 2 s, and the maximum stress on the wires was recorded.

**FIGURE 1:**
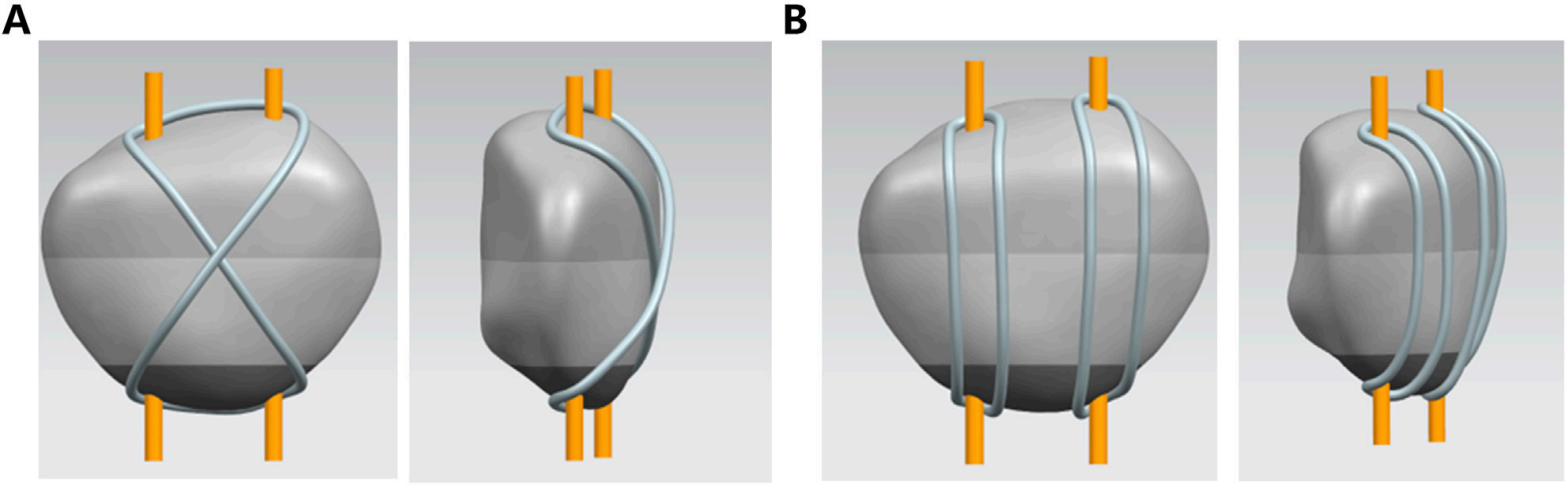
3d modeling of two internal fixation modalities. **(A)** Traditional single-tension band **(B)**. A dual-tension band.

Two comparison models were constructed: Group A used a traditional single tension band, and Group B used a dual tension band. The maximum stress on the wires was recorded for comparison (Figure 1).

### 2.2 Clinical cohort study

#### 2.2.1 Ethical approval and cohort description

This retrospective cohort study received ethical approval from the Ethics Committee. Informed consent was obtained from all participants. The study adhered to the World Medical Association’s Declaration of Helsinki. Inclusion criteria were: (1) surgically treated patellar fractures involving the articular surface and inferior pole within 14 days of injury; (2) age between 18 and 75 years. Exclusion criteria included: (1) concomitant fractures of the proximal tibia or distal femur; (2) severe knee function impairment before surgery (VAS >70 mm); (3) clinically diagnosed arthritis before surgery; (4) open patella fractures; (5) comorbidities such as uncontrollable diabetes, hypertension, malignancy, cardiovascular or cerebrovascular diseases, vascular diseases affecting the lower extremities, or any other conditions affecting lower limb functional assessment.

#### 2.2.2 Patients

From April 2021 to April 2023, 105 patients were enrolled in the cohort. This study analyzed 66 patients who underwent open reduction and internal fixation for patellar fractures involving the articular surface and inferior pole, with at least 1 year of follow-up. Surgeries were performed by experienced orthopedic trauma surgeons. Patients were divided into two groups based on their fixation method. Group A underwent ORIF with a traditional single tension band with cerclage fixation, while Group B used a dual tension band according to Deng’s surgical procedures (Zhang et al., 2020). Ring pins were allowed to be used in place of Kirschner pins in order to minimize implants migration, and additional screws or Kirschner wires could be used for complex fractures.

#### 2.2.3 Baseline data collection

**FIGURE 2:**
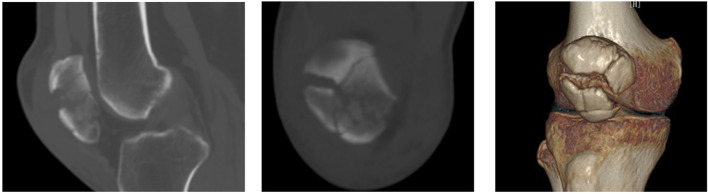
CT imaging of a patellar fracture involving the articular surface and inferior pole of the patella.

Preoperative data and patient information, including gender, age, and affected side, were recorded. Patellar fractures involving the articular surface and inferior pole were independently assessed by two senior orthopedic surgeons based on CT scans (Figure 2).

#### 2.2.4 Outcome measurement

Functional outcomes were assessed using the range of motion (ROM) and the Böstman score at each follow-up point. Complications were evaluated and treated as needed. At the final follow-up (1 year post-surgery), the Insall-Salvati Index (ISI) was measured on lateral knee radiographs with the knee in a 30° flexed position. Patella baja was defined as an ISI of less than 0.8 (Blackburne and Peel, 1977). Two researchers independently assessed all outcomes, including ISI and ROM, without grouping information, and the final measurements were averaged.

#### 2.2.5 Statistical analysis

Continuous variables were presented as mean and standard deviation (SD), while categorical data were expressed as numbers and percentages. Differences between groups were assessed using the chi-square test or Fisher’s exact test. All statistical analyses were performed using IBM SPSS ver. 26.0 (IBM Corp., Armonk, NY, United States). Significance levels were set at 0.05.

## 3 Result

### 3.1 Finite element analysis

**FIGURE 3:**
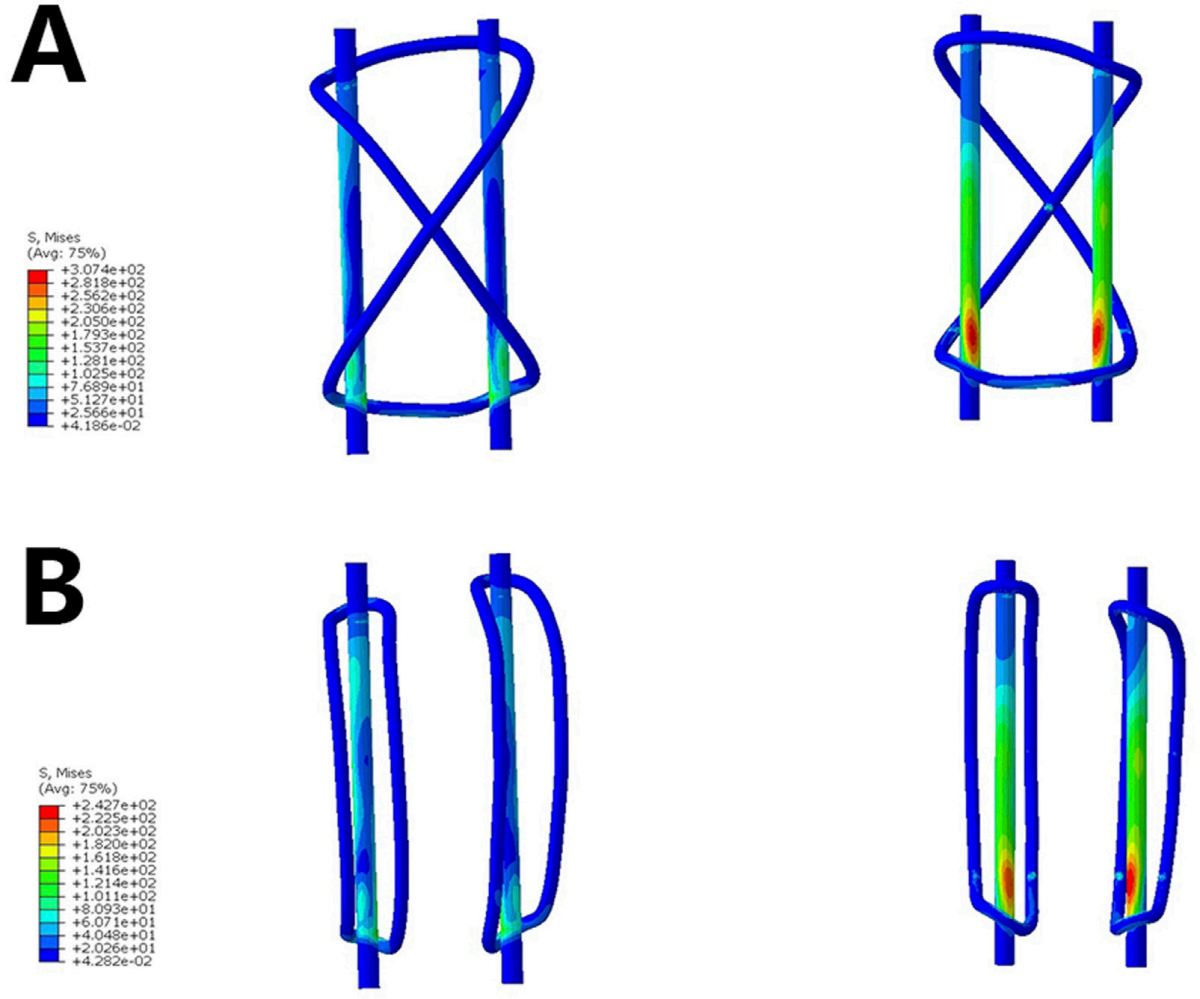
Stress on the internal fixation. **(A)**. Analysis of the stress on the single tension band **(B)** Analysis of the stress on the dual tension band.

A gradually increasing tensile load was applied to the superior pole of the patella’s 3D model of inferior pole fractures. At a tensile load of 500 N, the stress on the wires and patella was recorded. The results showed that the maximum patellar displacement in Group A was greater than in Group B (0.834 mm vs. 0.6 mm). The stress and displacement experienced by the Kirschner wires were also higher in Group A compared to Group B (307.4 MPa vs. 242.7 MPa, 0.849 mm vs. 0.601 mm) (Figure 3, Table 1).

**Table 1:** Analysis of patellar displacement and fixation stress.

	Group A	Group B
Maximum patellar displacement (mm)	0.834	0.6
Kirschner wire stress (MPa)	307.4	242.7
Kirschner wire displacement (mm)	0.849	0.601
Wire stress (MPa)	209	170.5
Wire displacement (mm)	0.831	0.574

**FIGURE 4:**
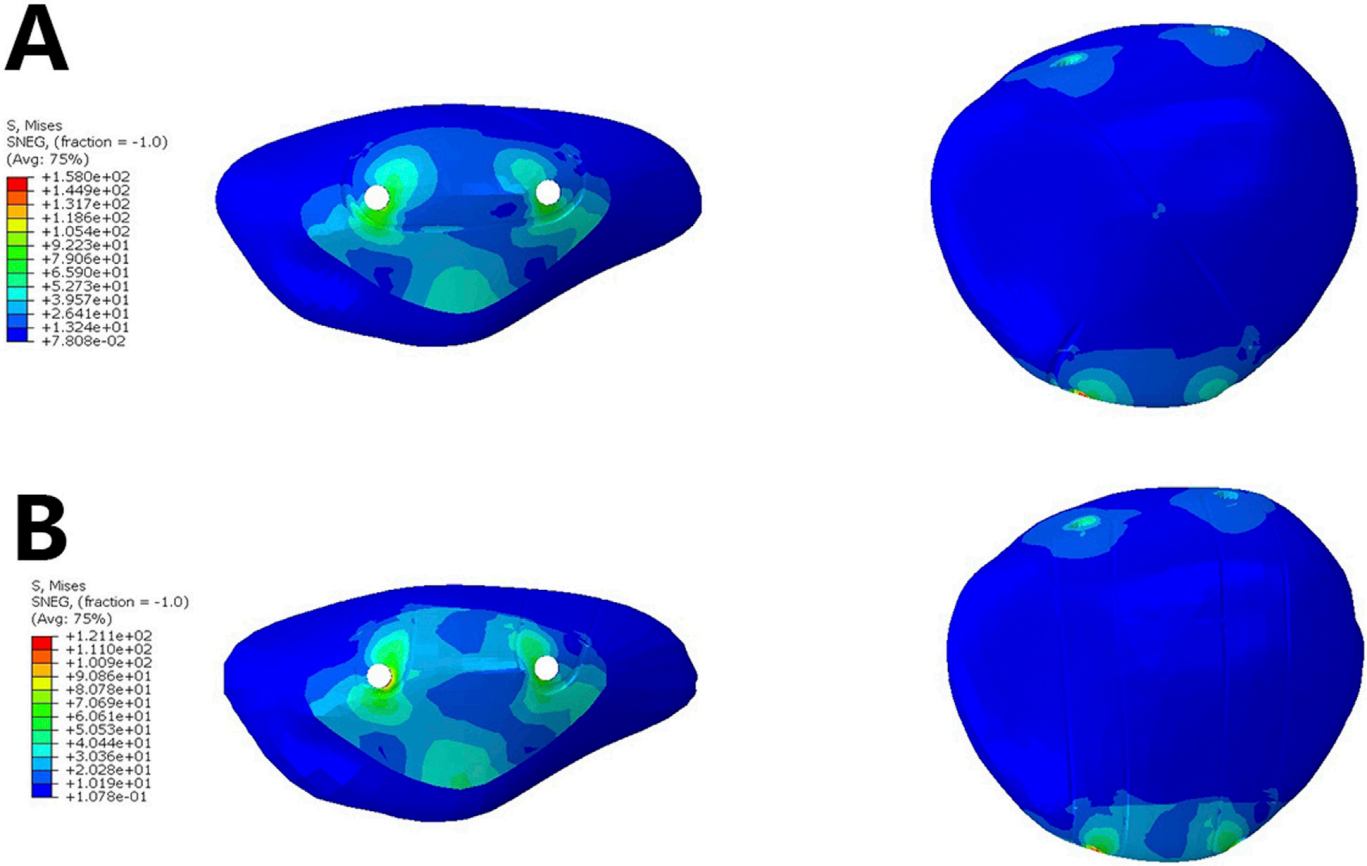
Stress on the Patella.- **(A)**. Analysis of the stress on the patella when using a single tension band **(B)** Analysis of the stress on the patella when using a dual tension band.

The analysis of the stress on the patella indicated that in Group A, the stress on the inferior pole of the patella was higher than in Group B (93.26 MPa vs. 76.79 MPa). This suggests that the dual tension band fixation helps to better distribute the concentrated stress on the inferior pole of the patella (Figure 4, Table 2).

**Table 2:** Analysis of stress on the Patella.

	Group A	Group B
Stress on the Patellar Superior Pole (MPa)	51.35	46.31
Stress on the Patellar Inferior Pole (MPa)	93.26	76.79

### 3.2 Clinical studies

#### 3.2.1 Patient cohort

**FIGURE 5:**
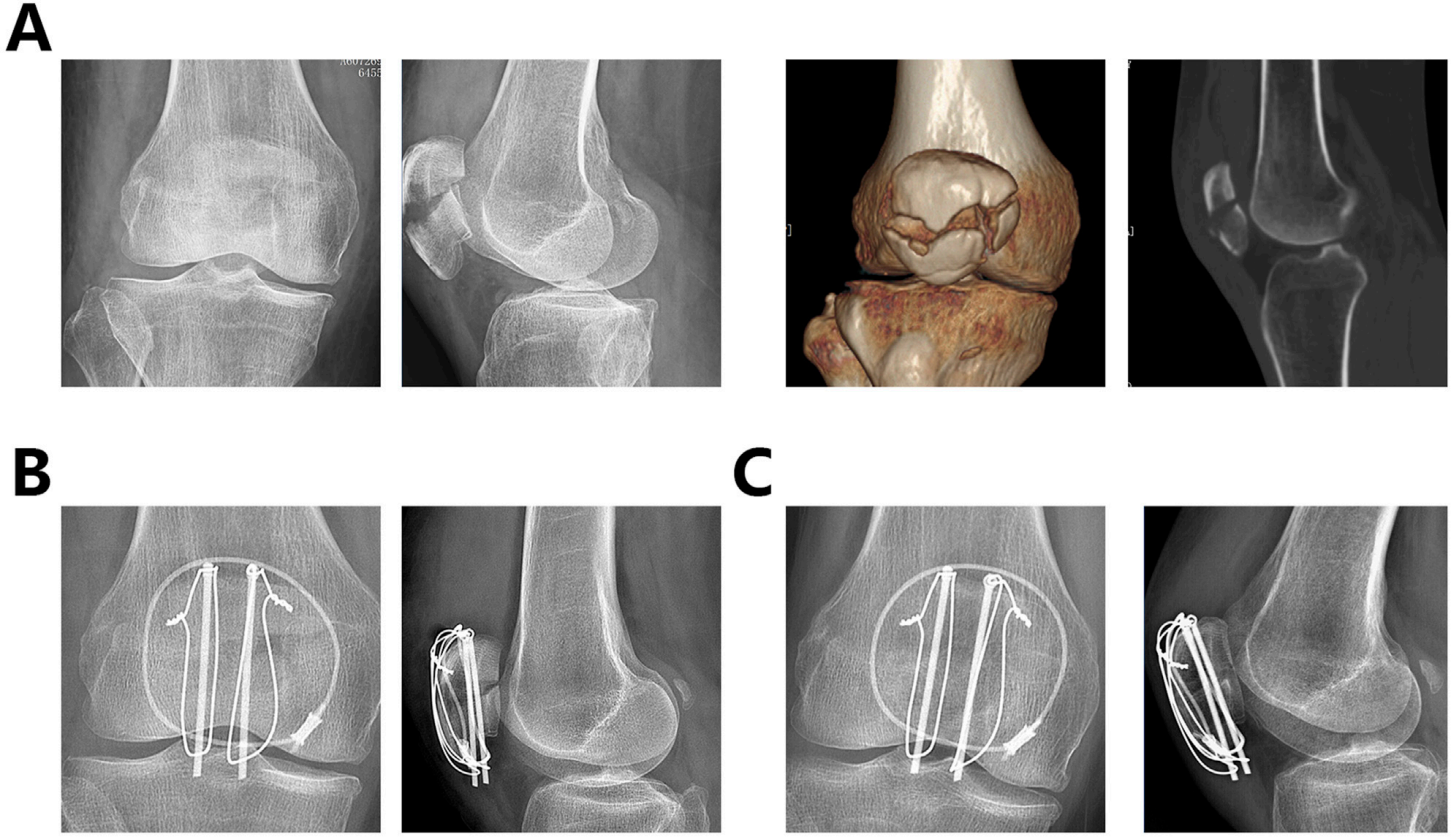
The radiographic images of a 67-year-old woman who underwent an internal fixation with the dual tension band technique. **(A)**. Preoperative knee radiographs, CT scans, and 3D reconstructions **(B)**. X-rays on the second postoperative day **(C)**. X-ray 1 year after surgery.

From April 2021 to April 2023, a total of 105 patients with patellar fractures involving the articular surface and inferior pole received surgical treatment at our hospital. After applying inclusion and exclusion criteria, 84 patients were eligible for the cohort. Excluding those who did not complete at least 1 year of follow-up, 66 patients were included in the final analysis. Of these, 30 (45.45%) were male and 36 (54.54%) were female. The age range was 32–74 years, with a mean age of 56.29 ± 11.51 years. Fractures were on the left side in 34 (51.51%) patients and on the right side in 32 (48.63%) patients. The mean follow-up duration was 14.323 ± 1.82 months. Fourteen additional K-wires or screws were used in group A and eleven in group B. Details of the patient groups are shown in Table 3. Figure 5 shows the radiographic images of a 67-year-old woman who underwent an internal fixation with the dual tension band technique, including preoperative CT and X-rays, postoperative X-rays the day after surgery, and 1 year postoperatively.

**Table 3:** Demographic data.

		Group A	Group B	Group C
Gender	Male	16	14	0.405
	Female	22	14	
Age		55.00 ± 11.01	68.04 ± 11.93	0.297
Affected side	Left	23	11	0.088
	Right	15	17	
Follow-up (mon)		14.65 ± 2.07	13.39 ± 1.14	0.108

#### 3.2.2 ISI and outcome

Table 4 provides an overview of ISI values, the occurrence of patella baja, range of motion (ROM), and postoperative Böstman functional scores. The results showed that Group B had significantly higher postoperative ISI values compared to Group A (0.93 ± 0.16 vs. 0.85 ± 0.17, p < 0.05). In Group A, patella baja occurred in 13 (34.21%) patients, whereas in Group B, it occurred in 7 (17.86%) patients. Group B also had better ROM (123.75 ± 9.58 vs. 117.63 ± 12.28, p < 0.05). The Böstman scores did not show a statistically significant difference (26.32 ± 2.19 vs. 25.76 ± 2.48, P = 0.347).

**Table 4:** ISI and outcome.

	Group A	Group B	P
ISI	0.85 ± 0.17	0.93 ± 0.16	0.034
Patella Baja	13 (34.21%)	7 (17.86%)	0.421
ROM	117.63 ± 12.28	123.75 ± 9.58	0.032
Böstman score	25.76 ± 2.48	26.32 ± 2.19	0.347

#### 3.2.3 Complications

**FIGURE 6:**
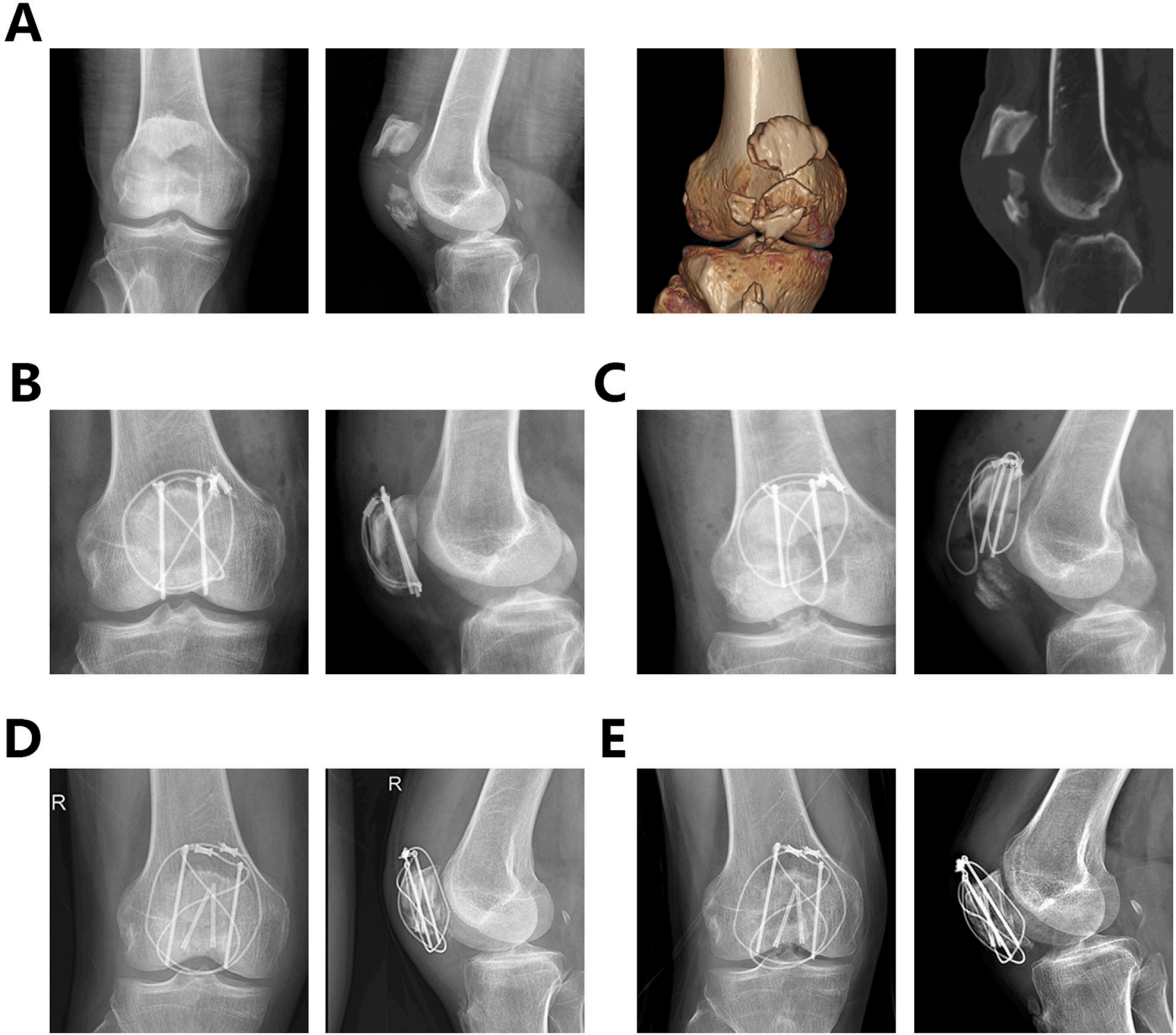
A 65-year-old male patient underwent internal fixation failure and revision surgery after being treated with a single tension band of internal fixation **(A)**. Preoperative x-rays and CT images **(B)**. Postoperative frontal and lateral views of the knee after the first surgery **(C)**. Postoperative frontal and lateral views of the knee at 2 months postoperatively showing failure of the internal fixation and displacement of the fracture **(D)** Postoperative frontal and lateral views of the knee after the second surgery **(E)**. Postoperative frontal and lateral views of the knee at one year postoperatively after the second surgery.

Among the 66 patients, one patient in Group B developed a postoperative superficial infection, which was resolved with dressing changes. One patient in Group A experienced fixation failure 2 months after the initial surgery and underwent a second internal fixation surgery (Figure 6). All patients achieved fracture healing without wire breakage.

## 4 Discussion

This study explores a novel internal fixation method for patellar fractures involving both the articular surface and the inferior pole. Through finite element analysis and retrospective research, we aim to demonstrate that this fixation method provides stable and effective immobilization while reducing the incidence of postoperative patella baja, thereby improving patient outcomes.

Numerous studies have reported instances of patella baja following fracture fixation, with prevalence rates ranging from 14.5% to 57%. However, this issue has not received adequate attention (Sebastian et al., 2022; Lazaro et al., 2013). Patella baja is closely associated with knee dysfunction and discomfort (Barth and Strickland, 2022). The positioning of the patella is intricately linked with increased stress complaints, as a lower patellar height can induce structural alterations in the patellar tendon and weaken the quadriceps muscle (Tecklenburg et al., 2006). Some studies have highlighted the correlation between inferior pole fractures and patella baja, but the specific causes remain unclear. These may be related to postoperative functional exercises or scar tissue formation, and further research is needed (Cutbill et al., 1997; Mariani et al., 1994; Lancourt and Cristini, 1975) Additionally, studies have noted other adverse effects, including joint stiffness, altered joint mechanics, decreased lever arm function, extensor lag, and reduced range of motion (Fox et al., 2012). Our previous research indicated that comminuted patellar fractures, in addition to inferior pole fractures, are significantly associated with postoperative patella baja. The “dual tension band” technique was introduced to address this challenge.

The Two-tension-band technique, introduced in 2016, has been employed for revision surgery to address fixation failure of patellar fractures. Researchers have suggested that this technique provides reliable internal fixation for patellar fractures (Xue et al., 2016). A wooden model study demonstrated that increasing the number of wires crossing the fracture line effectively enhances the pressure between fracture fragments, thereby promoting fracture healing (John et al., 2007). In 2019, Deng and colleagues applied the “dual tension band” technique to treat transverse or comminuted patellar fractures (Zhang et al., 2020). Their study concluded that this technique could effectively fix fractures involving the articular surface. However, no statistically significant differences were observed in long-term functional follow-up results. This might be attributed to the limited number of cases in the study, the lack of detailed classification and analysis of fracture types, and the authors’ primary focus on fracture healing without addressing postoperative patella baja.

For patellar fractures involving the lower pole, patellar height is a crucial prognostic indicator, as it can lead to restricted knee joint range of motion. While the mechanism behind this is not yet clear, we acknowledge that fracture type is indeed a contributing factor. Comminuted patellar fractures often result in early fixation failure. On the other hand, physicians may opt to extend the immobilization period for patients with comminuted fractures, which can indirectly limit knee function. In this study, the double tension band technique addresses the fixation of the fracture block in the inferior pole, but in clinical work patellar fractures are often comminuted. This leads to the fact that it is often unavoidable to add ring-tied wires to further improve the stability of internal patellar fixation.

The term “dual tension band” may not be entirely accurate, as this fixation method does not strictly adhere to the definition of tension bands. In this technique, a combination of Kirschner wires and wires creates a net-like structure that envelops the fractured inferior pole of the patella. The Kirschner wires stabilize the fracture fragments involving the articular surface, while multiple wires reduce stress concentration on the inferior pole and prevent anterior displacement of fracture fragments. Essentially, this method is closer to the Separated Vertical Wiring (SVW) technique, specifically adapted for inferior pole fractures. Unfortunately, vertical wiring alone does not adequately address intra-articular fracture fragments, and our technique fills this gap (Cho et al., 2018; He et al., 2018; Yan et al., 2021; Gao et al., 2023b). Clinically, our technique shows similar benefits to vertical wiring, reducing the incidence of postoperative patella baja in fractures involving the inferior pole.

Clinically, we found this fixation method applicable to various types of patellar fractures, including types A1, C1.2, and C1.3, which involve both the superior and inferior poles of the patella. It provides effective fixation while reducing surgical steps and saving operative time. For type C1.1 fractures, this method also offers effective fixation but does not show significant advantages over traditional tension band methods. These conclusions, however, require further systematic research for validation.

This study has several limitations. Implant removal is common in patients with patella baja, but due to the short follow-up period, we did not include data from patients who underwent implant removal. Due to the specific type of fracture, only 66 patients were enrolled in this study, but we believe that this study still proves that this type of internal fixation is an effective and reliable treatment. Finite element analysis cannot replace traditional biomechanical experiments; however, it can provide valuable guidance for clinical practice. The clinical problems we encounter are often more complex, involving soft tissue and various dynamic factors. Our research primarily validates the efficacy of this fixation method, and further large-scale prospective studies are needed to assess its overall effectiveness, including complications and functional outcomes.

## Data Availability

The original contributions presented in the study are included in the article/Supplementary Material, further inquiries can be directed to the corresponding authors.
