# Supplementary figures and images for: Dual tension band technique for patellar fractures involving articular surface and inferior pole: a retrospective cohort study and finite element analysis
Source: Front Bioeng Biotechnol. 2025 Mar 4;13:1530745. doi: 10.3389/fbioe.2025.1530745 (PMC11914795; doi:10.3389/fbioe.2025.1530745)

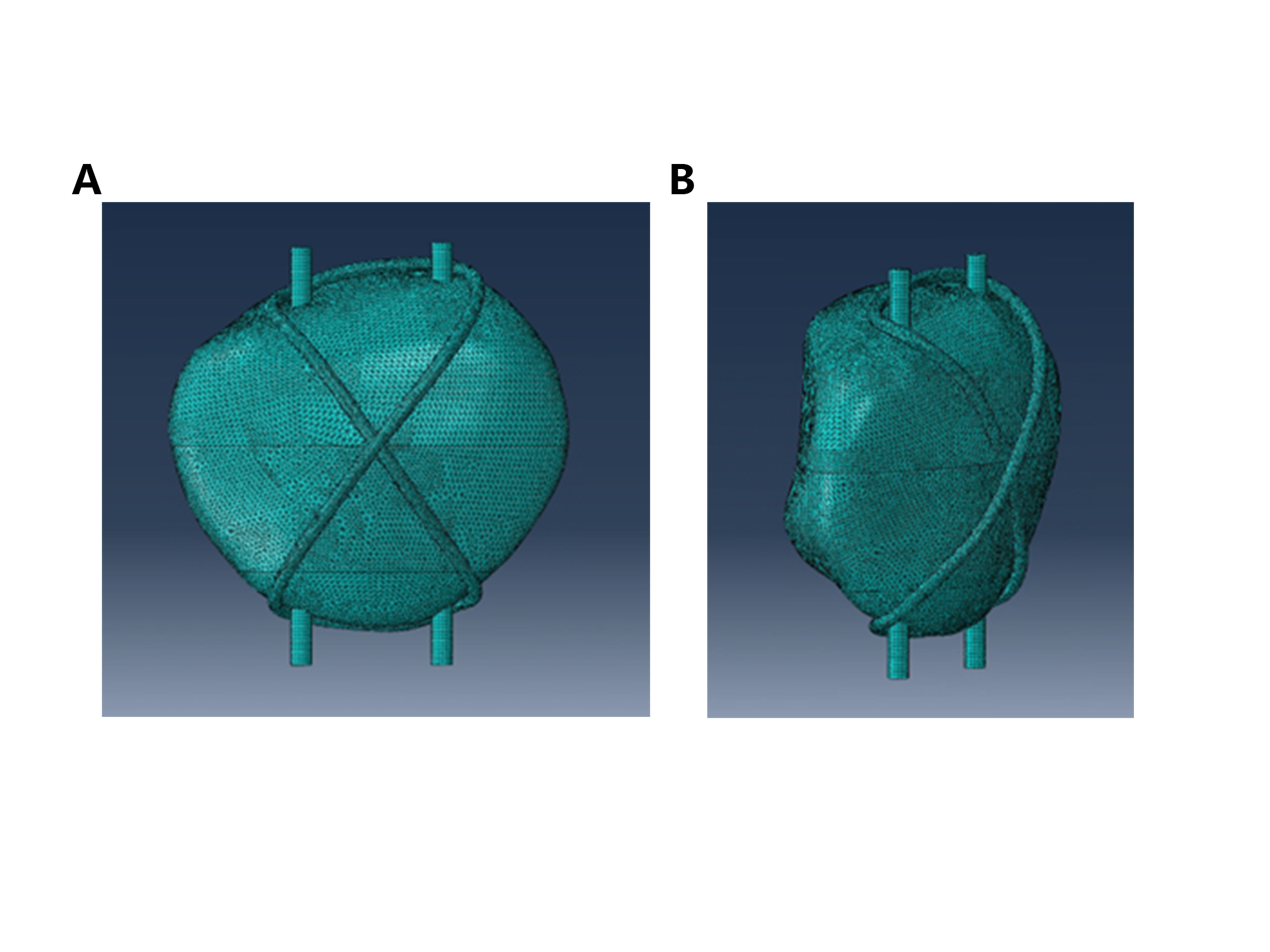

Supplement: Supplementary file 1 [file Image1.jpeg]
